# Prediction of the invasiveness of PTMC by a combination of ultrasound and the WNT10A gene

**DOI:** 10.3389/fendo.2022.1026059

**Published:** 2022-12-20

**Authors:** Zhang Yan, Liu Wen Gang, Guo Shi Yan, Ping Zhou

**Affiliations:** Department of Ultrasound, The Third Xiangya Hospital, Central South University, Changsha, China

**Keywords:** WNT10a, papillary thyroid microcarcinoma, ultrasound, invasion, prediction

## Abstract

**Objective:**

The purpose of this study was to predict the invasiveness of papillary thyroid microcarcinoma (PTMC) *via* ultrasonography in combination with the Wnt family member 10A (WNT10A) gene to provide a reference basis for evaluating the invasive capability of PTMC.

**Methods:**

Cancer tissue were collected from 182 patients with unifocal PTMC, and the patients were divided into the invasive group and the non-invasive group based on whether the lesions invaded the thyroid capsules or whether lymph node metastasis occurred. The expression of WNT10A protein was examined. Age, sex, maximum nodule diameter, color Doppler flow imaging (CDFI), nodule echo, microcalcification, aspect ratio, morphology (boundary), nodule location, internal structure, ultrasound-suspected lymph node metastasis (US-LNM), and WNT10A expression were compared between the invasive group and the non-invasive group. Univariate analysis and multivariate logistic regression analysis were performed, and a *p* value of less than 0.05 indicated that the difference was statistically significant.

**Results:**

(1) 36 patients in the non-invasive group showed high expression and 66 patients showed low or no expression, while 54 patients in the invasive group showed high expression and 26 patients showed low or no expression, suggesting that the expression level of WNT10A was higher in the invasive group than in the non-invasive group, with a statistically significant difference between the two groups (*P*<0.01). (2) Univariate analysis showed that there were statistically significant differences between the invasive PTMC group and the non-invasive group in age, sex, maximum nodule diameter, microcalcification, US-LNM and high WNT10A expression. (3) Multivariate analysis showed that the risk factors for invasiveness in patients with PTMC included age < 45 years, maximum nodule diameter > 7 mm, microcalcification, US-LNM and high WNT10A expression.

**Conclusion:**

The risk factors for PTMC invasiveness included age < 45 years, maximum nodule diameter >7 mm, microcalcification, US-LNM and high WNT10A expression. A combination of ultrasonography and WNT10A gene analysis could provide a reference basis for evaluating the invasive capability of PTMC.

## 1 Introduction

Thyroid cancer is an increasingly prevalent malignancy throughout the world, Papillary thyroid carcinoma (PTC) is the most common type of thyroid cancer (1, 2). Papillary thyroid microcarcinoma (PTMC) is defined as PTC measuring ≤1 cm, whether or not high-risk features such as lymph node metastasis and/or distant metastasis are present (3). PTMC may include two biological subgroups: high-risk PTMC which have high-risk features such as clinical node metastasis, distant metastasis, or significant extrathyroid extension such as to the trachea and recurrent laryngeal nerve, and low-risk PTMC without any high-risk features are generally an indolent disease, and most of them do not grow or they grow very slowly (3). For low-risk PTMC, active surveillance (AS) can be selected to avoid excessive treatment. For high-risk PTMC, active surgery and radioiodine therapy should be adopted. Some scholars have found that the incidence of LNM in patients with PTMC is between 4% and 65%, and the local recurrence rate is as high as 20% (4, 5). Therefore, it is important to accurately evaluate the risk stratification, which is related to the choice of treatment and the prognosis of PTMC patients.

Molecular diagnostic technology has become a hot spot and is widely used in auxiliary diagnosis of thyroid carcinoma (TC), which can make up for the deficiency of imaging examination and avoid excessive treatment of TC patients. At present, BRAFV600E gene is more studied. Li et al (6) think BRAFV600E mutation may contribute to risk stratification and management of PTMC. However, Barbaro et (7) found that the BRAF V600E mutation in papillary thyroid cancer with positive or suspected pre-surgical cytological finding is not associated with advanced stages or worse prognosis. The BRAF V600E mutation doesn’t appear to be a reliable risk factor for the aggressiveness of a tumor. BRAF analysis should neither be the only guide for pre-surgical decisions regarding the extent of surgery nor for post-surgical decisions regarding the aggressiveness of the treatment. The use of BRAFV600E alone may was not sufficient to accurately classify the risk of PTMC patients. Therefore, it is particularly important to find an effective molecular marker to increase the accuracy of PTMC diagnosis and risk stratification.

The research group searched the GEPIA database and found that WNT10A was correlated with the survival prognosis of patients with thyroid cancer. WNT10A is a member of the WNT gene family, which is clustered in the 2q35 region on human chromosomes (8). It has been confirmed that WNT10A mutation plays an important role in tooth dysplasia and ectoderm dysplasia (9–11), and WNT10A is highly expressed in a variety of malignant tumors, such as thyroid cancer, ovarian cancer, esophageal squamous cell carcinoma, renal cell carcinoma, and colorectal cancer (12–16). Dong et al (12) indicated that WNT10A may plays a crucial role in carcinogenesis and aggressiveness in papillary thyroid cancer by activating β-catenin-dependent pathway. There is no report on the combination of ultrasound, WNT10A and PTMC, and this study aimed to predict the invasiveness of PTMC *via* a combination of ultrasound and the WNT10A gene, establish a combined prediction model, and increase the accuracy of PTMC risk stratification. This study intended to provide the basis for the selection of clinical treatment strategies.

## 2 Materials and methods

### 2.1 Research subjects

This study was approved by the ethics committee of the hospital, and informed consent was obtained from all patients. The subjects of this study were patients who met the following requirements between December 2020 and December 2021: having received routine ultrasound examination before surgery; having undergone thyroidectomy and central lymph node dissection in our hospital (some patients also underwent cervical lateral lymph node dissection); and being diagnosed with unifocal PTMC by postoperative pathological examination. The exclusion criteria were as follows: PTMC patients with multiple nodules who did not undergo lymph node dissection, patients who had a history of cervical radiation therapy, and patients who had a history of neck tumor surgery. According to the pathological results, the patients were divided into invasive group and non-invasive group depend on whether the lesions invaded the thyroid capsules or whether lymph node metastasis occurred.

### 2.2 Instruments and examination methods

#### 2.2.1 Routine ultrasound examination

The instrument used in this study was the Acuson Sequoia Diagnostic Color Doppler Ultrasound System (Siemens, Mountain View, CA, USA). The probe frequency was 9-14 MHz. All sections of the thyroid gland were carefully scanned and the ultrasound characteristics of the thyroid nodules were observed and recorded, including the specific location of cancer foci, the size of cancer foci, the blood flow, the presence or absence of microcalcification, the aspect ratio (whether it was greater than 1), the number of nodules, and the infiltration of the capsule. In addition, cervical lymph nodes were also scanned. The criteria for ultrasound-suspected cervical lymph node metastasis (US-LNM) were as follows: a lymph node aspect ratio of > 0.5, the disappearance of lymphatic hilum structure, an unclear boundary between the cortex and medulla, cystic change, calcification, hyper-echo-genicity, and peripheral vascularization (17).

#### 2.2.2 Examination of the differential expression of WNT10A protein in PTMC by quantitative reverse transcription polymerase chain reaction

PTMC tissues were excised, lysed and ground. After centrifugation, the supernatants were collected. Reverse transcription primers were prepared. Finally, the reverse transcription primers and total RNA were transferred into PCR tubes and centrifuged. The mixture was prepared according to the proportions specified in the table below. The resulting reaction system was carried out in a water bath to obtain the reverse transcription product cRNA. The product was subjected to qRT-PCR analysis.

**Table:** 

Reagent	Volume added to each tube
M200 U/µL –MLV reverse transcriptase	0.5 µL
10 mM guanine triphosphate deoxynucleotide	2 µL
5X reverse transcription buffer	5 µL
40 U/µL RNA inhibitory enzyme	0.5 µL
RNase free water	6 µL

#### 2.2.3 Immunohistochemistry

The tissues submitted for examination were sectioned, baked, deparaffinized, hydrated, and washed. After blocking at room temperature for 10 min, the sections were subjected to antigen retrieval and then cooled to room temperature. The sections were then rinsed, blocked, and incubated overnight. The secondary antibody was then added. After washing and staining, the sections were dehydrated, mounted, sealed, and air-dried. The results were then judged by two pathologists who had worked in our hospital for more than ten years. If the two pathologists had different opinions on a case, a third doctor would participate in the discussion to reach the final judgment. The judgment criteria were as follows: ≥++, high expression; +, low expression; and -, no expression.

#### 2.2.4 Statistical methods

Statistical analysis was performed using SPSS 25.0 statistical software. The count data were expressed using frequency or rate, while the comparison between groups was performed using chi-square test. In the univariate analysis, the clinical indicators with *p*<0.05 were used as independent variables, while the dependent variable was the presence or absence of invasiveness in PTMC patients. After multivariate logistic regression analysis, the multivariate results were subjected to weight analysis using standardized regression coefficients. A *p* value of less than 0.05 indicated that the difference was statistically significant.

## 3 Results

### 3.1 General information

This study included 182 patients with unifocal PTMC (110 females and 72 males). The patients were aged between 21 and 69 years, with an average of 41.13 ± 6.21 years. The maximum diameter of nodules ranged from 4.5 to 10 mm, with an average of 7.86 ± 0.36 mm. In 88 patients, the diameter of nodules exceeded 7 mm. There were 80 patients in the invasive group and 102 patients in the non-invasive group.

### 3.2 Expression of WNT10A in the invasive group and non-invasive group

**Figure 1:**
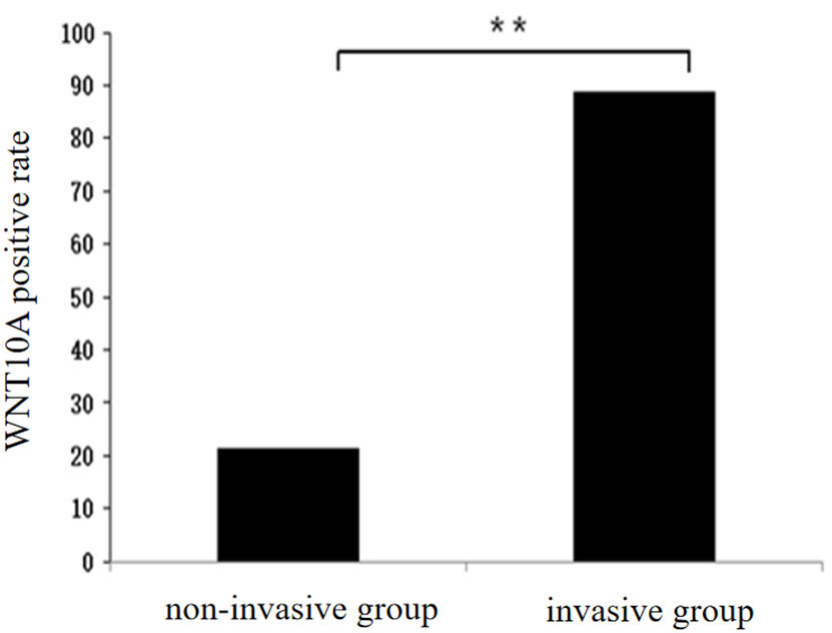
Differential expression of WNT10A in invasive group and non-invasive group (**P < 0.01).

36 patients in the non-invasive group showed high expression and 66 patients showed low or no expression, while 54 patients in the invasive group showed high expression and 26 patients showed low or no expression, suggesting that the expression level of WNT10A was higher in the invasive group than in the non-invasive group, with a statistically significant difference between the two groups (*P*<0.01, **Figure 1**).

### 3.3 Results of univariate analysis of the invasive group and the non-invasive group

The results showed that there were significant differences between the invasive group and the non-invasive group in age, sex, maximum nodule diameter, microcalcification, US-LNM, and high WNT10A expression (*p*<0.05). In particular, significant differences existed in US-LNM and high WNT10A expression (*p*<0.001). In contrast, there were no significant differences in CDFI, nodule echo, morphology (boundary), location, internal structure, or aspect ratio (*p*>0.05, **Table 1**).

**Table 1:** Univariate analysis of the invasive group and the non-invasive group.

Variable	Non-invasive group	Invasive group	x^2^	*p*
Age (years)				
<45	42 (41.2%)	53 (66.3%)	11.297	0.001
≥45	60 (58.8%)	27 (33.7%)		
Gender			4.123	0.042
Male	47 (46.1%)	25 (31.3%)		
Female	55 (53.9%)	55 (68.7%)		
Maximum diameter of nodule (mm)			9.510	0.002
>7	39 (38.2%)	49 (61.3%)		
≤7	63 (61.8%)	31 (38.7%)		
CDFI			1.680	0.641
Absence	19 (18.6%)	10 (12.5%)		
Peripheral type	21 (20.6%)	20 (25.0%)		
Internal type	30 (29.4%)	22 (27.5%)		
Mixed type	32 (31.4%)	28 (35.0%)		
Nodule echo			0.253	0.615
Very hypoechoic echo	42 (41.2%)	30 (37.5%)		
Other echoes	60 (58.8%)	50 (62.5%)		
Microcalcification			5.703	0.017
Presence	38 (37.3%)	44 (55.0%)		
Absence	64 (62.7%)	36 (45.0%)		
Aspect ratio			0.099	0.753
>1	38 (37.3%)	28 (35.0%)		
≤ 1	64 (62.7%)	52 (65.0%)		
Morphology (boundary)			2.526	0.112
Regular	39 (38.2%)	40 (50.0%)		
Irregular	63 (61.8%)	40 (50.0%)		
Nodule location			0.353	0.950
Upper pole	22 (21.6%)	20 (25.0%)		
Middle pole	48 (47.1%)	37 (46.3%)		
Lower pole	26 (25.5%)	19 (23.8%)		
Thyroid isthmus	6 (5.9%)	4 (3.9%)		
Internal structure			2.160	0.142
Solidity	73 (71.6%)	49 (61.3%)		
Non-solidity	29 (28.4%)	31 (38.7%)		
WNT10A expression			18.604	<0.001
High expression	36 (35.3%)	54 (67.5%)		
Low or no expression	66 (64.7%)	26 (32.5%)		
US-LNM			5.710	0.017
Yes	24 (23.5%)	32 (40.0%)		
No	78 (76.5%)	48 (60.0%)		

### 3.4 Results of multivariate analysis of the invasive group and the non-invasive group

**Figure 2:**
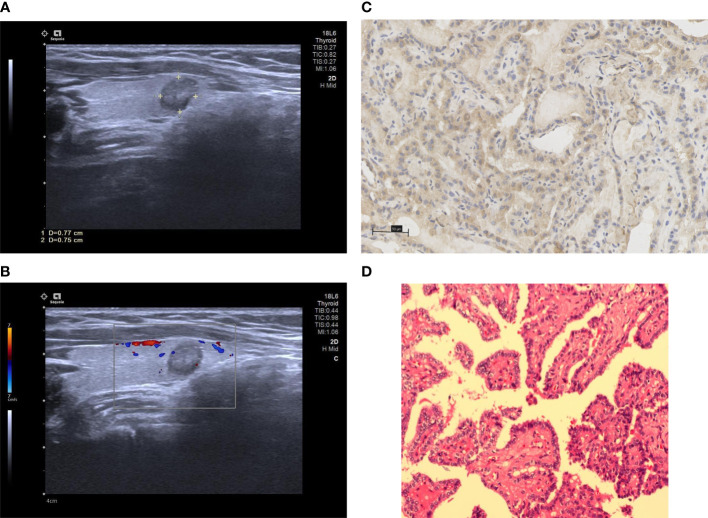
The above images were from a 28-year-old female patient. **(A)** a conventional two-dimensional image. The nodule was 7.7 mm x 7.5 mm in size and contained microcalcifications. **(B)** CDFI of the nodule, which only showed a few punctate blood flow signals. **(C)** the high expression of WNT10A. **(D)** pathological sections of the lesion indicating PTMC.

**Figure 3:**
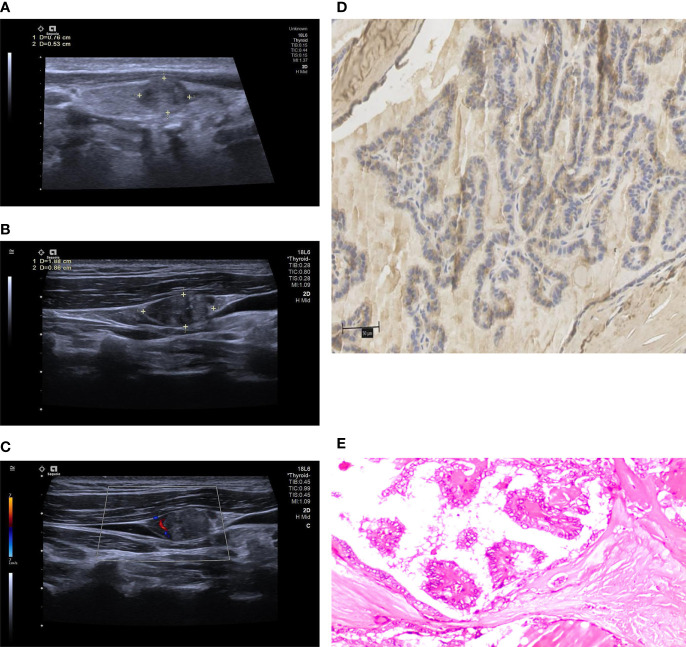
The above images were from a 40-year-old male patient. **(A)** a conventional two-dimensional image. The nodule was 7.6 mm x 5.3 mm in size. **(B)** the image of ipsilateral cervical lymph node. The size of the lymph node was 18.8 mm x 8.6 mm. The lymphatic hilum structure disappeared, and the boundary between cortex and medulla was unclear. All the characteristics were conformed to US-LNM. **(C)** CDFI of the lymph node, which showed dotted or linear blood flow signals. **(D)** the high expression of WNT10A. **(E)** pathological sections of the lesion indicating PTMC.

Multivariate analysis was performed on the indices that had a *p* value of less than 0.05 in the univariate analysis to verify whether they were risk factors for invasiveness in patients with PTMC. The following risk factors for invasiveness in PTMC patients were identified: age < 45 years (OR=2.125, *p*=0.031), maximum nodule diameter > 7 mm (OR=2.353, *p*=0.014), microcalcification (OR=2.371, *p*=0.016), US-LNM (OR=2.418, *p*=0.023) and high WNT10A expression (OR=3.610, *p*<0.001, **Table 2**; **Figures 2**, **3**).

**Table 2:** The results of multivariate analysis.

Variable	Results of multivariate analysis		
	OR	*p*	β
Age	2.125 (1.069-4.224)	0.754	0.031
Gender	0.513 (.254-1.037)	-0.667	0.063
Maximum diameter of nodule	2.353 (1.190-4.654)	0.856	0.014
Microcalcification	2.371 (1.175-4.781)	0.863	0.016
US-LNM	2.418 (1.129-5.175)	0.883	0.023
WNT10A gene	3.610 (1.810-7.203)	1.284	<0.001

### 3.5 Receiver operating characteristic curve analysis

**Figure 4:**
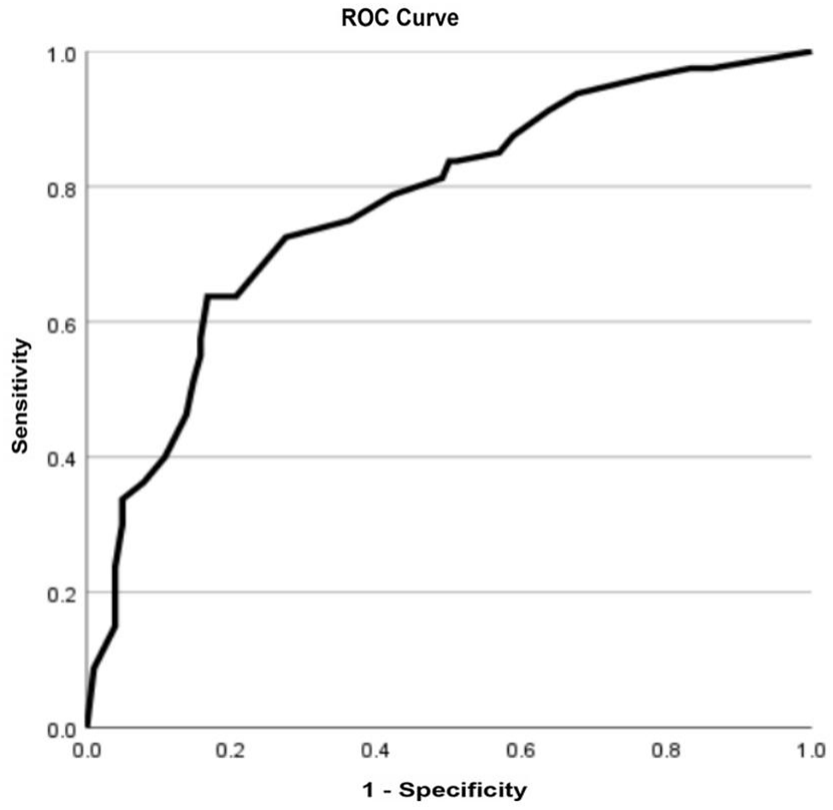
The ROC curve.

ROC curves were plotted using the invasiveness as the ordinate. The abscissa of the ROC curves were age < 45 years, maximum nodule diameter > 7 mm, microcalcification, US-LNM and high expression of WNT10A. The efficacy of the ROC curves in predicting the invasiveness of PTMC was evaluated. The area under the curve was 0.771, the sensitivity was 72.5%, and the specificity was 72.5% (**Figure 4**).

## 4 Discussion

Our previous study showed that malignant thyroid nodules could be effectively diagnosed based on five ultrasonic signs, including solidity, aspect ratio >1, irregular border, microcalcification, and hypoechoic or very hypoechoic appearance (18). However, this study found that only microcalcifications showed a significant difference in regard to the invasiveness of PTMC. Calcification originated from necrotic cancer cells in lymphatic vessels. Therefore, microcalcification might be related to lymph node metastasis, which was consistent with the results of Jeon et al (19). The aspect ratio > 1 was a typical malignant sign of thyroid cancer, but its correlation with the invasiveness of PTMC was not observed in this study. Ultrasonography could effectively examine lymph nodes in regions I, II, III, IV, and V. Clinically, great attention should be given when US-LNM is found. However, ultrasonography showed a low sensitivity toward the lymph nodes in zone VI, which was due to the obstruction of the trachea and esophagus causing increased difficulty of detection. There is no significant correlation between the amount of color Doppler signal and invasiveness, which may be due to the fact that blood vessels in malignant nodules, angiogenesis cannot keep up with the rapid growth of tumors, resulting in a large number of vascular necrosis in the lesions. Therefore, it is difficult to detect color Doppler signal in PTMC (20), no matter in the invasive group and non-invasive group.

In this study, we found that an age of less than 45 years was another important risk factor for the invasiveness of PTMC. Sun et al. also believe that there is a significant correlation between an age of less than 45 years and a high risk of LNM (21, 22). In addition, some scholars believe that patients younger than 40 years have an increased risk of invasion (23). Although the incidence of PTMC is higher in women than in men, this study did not find that PTMC was more likely to develop invasiveness in female patients. Some scholars suggest that men are an important risk factor for aggressive behavior, which may be associated with unhealthy lifestyles, such as smoking and drinking (21, 22).The size of PTMC nodules had an impact on the probability of invasiveness. The risk of LNM increased when the nodule was greater than 7 mm in diameter, which could serve as an important predictor of LNM. Some scholars also believed that the larger the nodule was, the higher the probability of invasiveness (24–26). Gur et (27, 28) proposed that the formation of multiple nodules was related to an increased risk of local recurrence, lymph node metastasis and long-distance metastasis. They believe that the formation of multiple nodules is an adverse predictor of papillary thyroid cancer (PTC) and might be related to tumor clonal selection and thyroid cancer spread. This study did not include patients with multifocal PTMC because it was difficult to determine which nodule developed invasiveness. To avoid bias, we only selected patients with single lesions.

It has been demonstrated that WNT10A is closely related to PTC, and activation of the WNT10A/β-catenin signaling pathway promotes cell proliferation and migration (12). Clinicopathological association analysis revealed that Wnt10a was significantly associated with high-grade and late-stage ovarian cancer, which suggested that Wnt10a serves an oncogenic role during the carcinogenesis and progression of ovarian cancer (13). Long et al (14) have found that high WNT10A expression levels correlate with poor survival. WNT10A siRNA knockdown decreased cell proliferation and aggressiveness of renal cell carcinoma (RCC), and WNT10A acts as an autocrine oncogene both in RCC carcinogenesis and progression by activating WNT/β-catenin signaling (15). Our study showed that PTMC patients with high WNT10A expression had an increased risk of invasion and the odds ratio (OR) was 3.610. Such findings indicate that WNT10A promoted the migration and invasion of PTMC. We will further study the mechanism of WNT10A promoting PTMC cell invasion from the cytological and zoological aspects.

In summary, age of less than 45 years, maximum nodule diameter greater than 7 mm, microcalcification, US-LNM and high expression of WNT10A were risk factors for PTMC invasion. This study had certain limitations: (1) The accuracy and stability of this risk model were not verified. (2) Elastography and contrast-enhanced ultrasound were not included to jointly evaluate the invasiveness of PTMC. This study is a retrospective study of postoperative specimens of patients. In the future, we will try to establish a pre-operative risk model by measuring WNT10A mRNA levels in the eluate of patients’ pre-operative FNAB samples by RT-PCR, and verify the accuracy and stability of the model after surgery.

## Data availability statement

The original contributions presented in the study are included in the article/supplementary material. Further inquiries can be directed to the corresponding author.

## Ethics statement

The studies involving human participants were reviewed and approved by The IRB of Third Xiangya Hospital, Central South University. The patients/participants provided their written informed consent to participate in this study.

## Author contributions

ZY did the first draft. PZ provided instructive advice and useful suggestions for this manuscript. LG and GY provided statistical advice for this manuscript. All authors contributed to the article and approved the submitted version.
